# ‘It puts life in us and we feel big’: shifts in the local health care system during the introduction of rapid diagnostic tests for malaria into drug shops in Uganda

**DOI:** 10.1080/09581596.2014.886762

**Published:** 2014-03-03

**Authors:** Eleanor Hutchinson, Clare Chandler, Siân Clarke, Sham Lal, Pascal Magnussen, Miriam Kayendeke, Christine Nabirye, James Kizito, Anthony Mbonye

**Affiliations:** ^a^ Department of Global Health and Development, London School of Hygiene and Tropical Medicine, London, UK; ^b^ Department of Disease Control, London School of Hygiene and Tropical Medicine**,**London, UK; ^c^ Institute for International Health, Immunology and Microbiology, Centre for Medical Parasitology & Institute for Veterinary Disease Biology, Section for Parasitology and Aquatic Diseases, University of Copenhagen, Copenhagen, Denmark; ^d^ The ACT 2 Project, Kampala, Uganda; ^e^ Department of Community Health, Ministry of Health, Kampala, Uganda

**Keywords:** Malaria, rapid diagnostic tests, private sector, complex intervention trials, health systems

## Abstract

This paper is an analysis of the social interaction between drug sellers, their clients and local health care workers within a medical trial that introduced rapid diagnostic tests for malaria into private sector drug shops in Mukono District, Uganda. It locates the introduction of a new technology to test blood and a system of referral within the context of local concerns about the choice and evaluation of treatment; and the socially legitimated statuses, roles and hierarchies within the local health care system. Based on the multi-layered interpretation of 21 focus group discussions, we describe three key aspects of the trial central to local interpretation: openly testing blood, supervisory visits to drug shops and a new referral form. Each had the potential to shift drug shop vendors from outsider to insider of the formal health service. The responses of the different groups of participants reflect their situation within the health care system. The clients and patients welcomed the local availability of new diagnostic technology and the apparent involvement of the government in securing good quality health services for them from providers with often uncertain credentials. The drug shop vendors welcomed the authorization to openly test blood, enabling the demonstration of a new skill and newfound legitimacy as a health worker rather than simple drug seller. Formal sector health workers were less enthusiastic about the trial, raising concerns about professional hierarchies and the maintenance of a boundary around the formal health service to ensure the exclusion of those they considered untrained, unprofessional and untrustworthy personnel.

## Introduction

Over the last 20 years, with the growth of private sector medicine across sub-Saharan Africa, debates about its benefits, potential and dangers have become commonplace among global and national health policy-makers (Basu, Andrews, Kishore, Panjabi, & Stuckler, 2012; Fraser & Druce, 2006; Hozumi et al., 2009). In the context of poorly funded and often fractured public health services, the notion of ‘harnessing’ informal bio-medical providers to improve access to medication at community level has become a particularly attractive option (Arrow, Panosian, & Gelband, 2004; Basu et al., 2012; Forsberg, Montagu, & Sundewall, 2011; Fraser & Druce, 2006). Attempts to utilize this supply chain and improve access to treatment and quality of service provision have been made with varying success, most notably of late through the introduction of the Affordable Medicines Facility for malaria (Tougher et al., 2012).

Anthropological approaches to the analysis of the roles of informal providers within health systems offer the opportunity to extend and provide nuance to these policy debates about drug and diagnostic availability, affordability and potential for scale-up in the informal private sector (Chandler et al., 2011; Cross & MacGregor, 2010; Geest, 1999; Kleinman, 1980; Pinto, 2004; Reynolds Whyte, 1991; Reynolds Whyte, Geest, & Hardon, 2002; Whyte, 1992). Concerns with the intersections between bio-medical knowledge and power has led anthropologists to examine the multiple ways in which informal bio-medical providers exist in relation and reference to the (often publically funded) formal system but ultimately operate beyond its authority (Cross & MacGregor, 2010; Geest, 1999; Kleinman, 1980; Whyte, 1992). Informal providers, who already occupy other positions of power within their community, have been shown to be highly successful at utilizing language and bio-medical material culture to more or less continuously ‘borrow’ institutional bio-medical legitimacy (Mogensen, 2005; Pinto, 2004). Others, in common with many marginal actors, have been shown to occupy a social role with a fluctuating duality and inherent contradiction, shifting in and out of the formal system with the ebb and flow of global and national policy-making (Chandler et al., 2011; Cross & MacGregor, 2010; Pinto, 2004). The literature suggests that interventions to make improvements to the services offered by these informal providers (even apparently simple technological innovations such as rapid diagnostic tests (RDTs) for malaria) will be made complex and have multiple effects as they are refracted through intricate social and institutional relationships.

This paper is concerned with registered drug shop vendors who occupy an official role in the formal Ugandan health system but who in practice also operate as informal drug sellers providing services beyond the parameters of their license and registration (Chandler et al., 2011; Cohen et al., 2012; Whyte, 1992). Here, we examine a moment when the relationship between these drug shop vendors and the formal health system appeared to shift as they became the subjects of a research trial (‘the project’ hereafter) that introduced RDTs for malaria into registered drug shops for a period of 18 months. The case is useful for the analysis of the response of marginal actors to the introduction of a new health innovation. It also offers insights into the potential impacts of the scale-up of RDTs in this setting, a policy option that is coming under increasing scrutiny by national governments and the global policy community.

Based on social science research carried out alongside the main project activities, this paper focuses on its largely undeclared intentions by examining the intervention through a health systems lens. Such an approach foregrounds the notion that health care is sought, managed and governed in different interconnected sites, and that institutional and individual action in one site shifts the context in which other social actors operate (Smith & Hansen, 2012). From an anthropological perspective, it also requires the understanding that social activity surrounding the seeking and provision of health care occur within more or less connected constellations of social relationships, institutions and material culture (Kleinman, 1980). As elements of social life, the types of relationships, resources available, their uses and the interpretation of their significance are fluid and shift over time, shaping and being shaped by (amongst other forces) public health interventions that seek to modify treatment-seeking and medical practice.

The paper uses the data to draw the reader through the processes of the project from the perspective of each group of participants, the drug shop vendors, the clients and the health workers from government and mission health-centres nearby. It gives the background to the position of drug shops within the Ugandan health system linking this to the response of the drug shop vendors as they were offered the opportunity to join the project and were provided with training, materials and supervisory visits from the project staff. The second half of the paper revolves around an analysis of the production of the social meaning of the project by focusing on the ways in which the research participants interacted with the main research objects. Maintaining a theoretical distinction between subjects and objects, we explore how the rapid diagnostic test and the referral form was used and made sense of, in the particular context of the project and networks of relationships. In the context of the health system in Uganda, we argue that the use of these objects by the research participants had the power to transform the drug shop vendors into a legitimate bio-medical practitioner in the local community, while, at local health facilities, permitting the explicit denial of their entry into the formal health service.

## The project

Between October 2010 and July 2012, a cluster-randomized controlled trial to evaluate the cost-effectiveness and feasibility of introducing of RDTs in registered drug shops was conducted by the Ugandan Ministry of Health in collaboration with the University of Copenhagen and the London School of Hygiene and Tropical Medicine (www.actconsortium.org/RDTdrugshops). The project hypothesized that use of RDTs in the drug shops would reduce the over-diagnosis and over-prescription of anti-malarial drugs and thereby result in an increased proportion of patients receiving appropriate treatment for malaria as the primary outcome. The project recruited registered drug shop vendors and randomized them into two groups by diagnostic method: one received training in presumptive diagnosis of malaria and artemisinin combination therapies (ACTs) (the control arm); and the other received training in the use of RDTs for diagnosis of malaria and ACTs (the intervention arm). Both groups were provided with a three day training in the clinical symptoms of malaria (including severe disease) and other febrile illnesses, the use of ACTs (including rectal artesunate), referring patients to local health facilities and hospitals and patient communication. All drug shop vendors were trained to take a finger prick blood sample and prepare blood slides to provide the project’s outcome evaluation data. For those randomized into the RDT arm, an additional day of training was also provided in the use of RDTs and integration into practice. Following evaluation of their knowledge and skills at the end of the training, all drug shop vendors were provided with a certificate and returned to their shops with a supply of materials: ACTs; blood slides and slide box; gloves, lancets, swabs and cotton wool; treatment registers for record keeping and triplicate forms for record keeping of treatment dispensed and those requiring referral. RDTs were also provided for the drug shop vendors in the intervention arm, and a roadside sign to advertise the availability of diagnostic testing at that locality. All materials were supplied to the drug shop vendors free of charge. The workshop training was followed by a two-month period of supervision at the beginning of the intervention, during which each drug shop vendors received at least three supervisory visits by the project team. After this supervision was scaled back, but periodic contact was maintained when drug shop vendors visited the project office to replenish supplies. After the first 12 months of implementation, the project team again visited the drug shops and clients who had received treatment, as part of the evaluation.

## Methods

### Data collection

Fieldwork for the qualitative data collection was carried out by three Ugandan social scientists under the supervision of a London based anthropologist between November 2011 and February 2012. In all, 21 Focus Group Discussions (FGDs) were conducted with: drug shop vendors in the intervention arm (*n* = 4) and the control arm (*n* = 3); drug shop clients and local residents (members of the community hereafter) in the intervention arm (*n* = 4) and the control arm (*n* = 2); and with health worker staff from health facilities in the intervention area (*n* = 5) and the control area (*n* = 3). With agreement from participants, the focus groups were recorded on a digital recorder. Following each focus group a summary was written and sent to the anthropologist based in London and used as the basis for ongoing analysis and changes to questions asked in the field. Each focus group was transcribed into Luganda and then translated using meaning-based translation into English (Larson, 1998).

In the community focus groups, participants were resident in the study area and had been a client, or the carer of a client who had been the subject of a referral from a drug shop within the last year. Separate focus groups were held with community members who attended drug shops in the intervention and control arms. For the health unit staff focus groups, participants had to have been working at a health unit in the study area for more than six months. For the drug shop vendor focus groups, participants had been working at a drug shop in the study area for more than six months. The drug shop vendors were further stratified between those who referred most frequently, second most frequently, least frequently and not at all. Topic guides covered treatment-seeking behaviour, experiences at drug shops, experiences with RDTs and referral.

The drug shop vendors and members of the community were identified from project records. The majority of drug shop vendors and health worker focus groups were conducted in a basic, Sunday school structure that had benches for the participants to sit on but was otherwise unequipped. Two focus groups with health workers were conducted at their health facilities. Community focus groups were conducted in different venues within their respective communities. Participants received money to cover the cost of their transport and refreshments.

FGDs provide a particular kind of information. Despite claims about its naturalness as a setting for discussion and attempts by the facilitators to create a relaxed atmosphere, focus groups sit at the formal, public end of social life and are shaped by this belonging (Schensul, Lecompte, Nastasi, & Borgatti, 1999). Further, the people in our focus groups often interpreted the facilitators as being medically trained, belonging to the project and/or being medical personnel from the Ministry of Health. This positioning of the facilitators will have shaped the discussions by providing a particular focus for some of the participants who may have been seeking extra resources from project personnel or seeking to cover and deny their informal or illegal activities. Moreover, our interpretation of the significance of the project to its different constituencies is necessarily shaped by the experiential insight gained as the social scientists negotiated these relationships and were drawn into the power relationships inherent in this formal/informal system.

In addition to the focus groups, background information on the representation of drug shops and the drug shop vendors at national level was gathered. Using the terms ‘drug shop’ ‘private clinics’, ‘drug sellers’ and ‘private medicine’ the main government-controlled newspaper *New Vision* was searched for news stories for the year preceding the project (2009) until the end of 2012. Of the 100 news stories initially identified, 47 were considered to be relevant to the current study.

### Data analyses and ethical consideration

The coding of transcripts was carried out by three Ugandan social scientists working under supervision of an anthropologist. The initial coding scheme was drawn up through a combination of themes emerging from the data, pre-defined areas of interest identified from the main intervention and from anthropological theory. As far as possible, all the coding for each set of focus groups was carried out by a single social scientist using Nvivo qualitative data coding software with close support from the anthropologist. The main findings of the project were discussed with the anthropologist, principal investigator of the project and the project social scientists over the course of a week’s analysis meetings held at the Ministry of Health in Kampala. The anthropologist undertook the more conceptual level of analysis, revisiting and linking themes emerging in the coding of transcripts.

The newspaper stories for the discourse analysis were analysed by hand by the anthropologist focusing on the representation of the drug shop vendors, the construction of their identity and variety of ways in which they were positioned in relation to state authorities.

Ethical approval was obtained from the London School of Hygiene and Tropical Medicine and the Uganda National Council for Science and Technology.

## Drug shops in context

Uganda’s post-colonial health care system has been dominated by an interdependent formal public and informal private system of bio-medical care; and a high demand for western pharmaceuticals (Adome, Whyte, & Hardon, 1996). Bio-medical care is divided between state, non-governmental organizations, faith-based organizations and not-for-profit facilities; private hospitals and clinics; and medicine sellers who operate from drug shops, grocery stores and market places (Adome et al., 1996; Mogensen, 2005).

As in other developing countries, the boundaries between the different elements of the system are blurred with personnel, equipment and medication moving between for-profit and not-for-profit sectors (Bloom, Standing, & Lloyd, 2008). Staff from state hospitals and health-centres supplement their income selling drugs at drug shops and providing drugs for sale at health-centres. In the face of stock-outs, patients being cared for within government health-centres often purchase medicine and equipment from the private for-profit sector (Adome et al., 1996; Chandler et al., 2011; Mogensen, 2005). Yet, despite this fluidity, research shows that Ugandan patients and clients seek different types of services from different sectors of the health system. Although public sector health facilities often lack the drugs available in the private sector, these facilities are valued for their trained staff and diagnostic services. Moreover, the diagnostic examination in the health facility often ends with the results of the diagnosis being written on paper, an act that is powerfully symbolic of the expertise of the health worker (Mogensen, 2005). As Mogensen argues:

People know what they are suffering from, but examination, machines, and instruments (thermometers, stethoscopes, microscopes) help the health workers identify the physical entity, which the medicine, in turn, is supposed to work on. In private drug shops you can buy the medicine, actively participate in the decision about what and how much to buy, and even be treated in a friendly manner. But the problem, people add, is that ‘there they do not do the examination.’ (Mogensen, 2005)

Medicine sellers are overwhelmingly the most popular distributers of pharmaceuticals including anti-malarial medication and are very often the first point of call for those with febrile illness (Rutebemberwa, Pariyo, Peterson, Tomson, & Kallander, 2009). In most urban and some rural areas drug shops are ubiquitous and competition for clientele is intense (Cohen et al., 2012, Medicines and Health Services Delivery and Monitoring Unit (MHSDMU) 2011). A minority of the drug shops are formally registered and licensed by government to sell class C (over the counter medication) but the majority are unlicensed and may remain so for years at a time (MHSDMU, 2011). A combination of high demand for bio-medicine, frequent stock-outs at public health facilities and a lack of supervision and control also results in many of the formally registered drug shop vendors practicing medicine informally, providing antibiotics, filling prescriptions and giving injections (Adome et al., 1996; Eadsi, 2013; Reynolds Whyte, 1991; Whyte, 1992).

Earlier work by Reynolds Whyte on the social relationship between bio-medical drug sellers in Uganda and their clientele demonstrated that though many drug sellers belong to public sector institutions, when they worked as a drug seller, institutional belonging lost significance (Whyte, 1992). Reynolds White argues that Ugandan drug shop vendors gained legitimacy from the community, and authority from their ongoing demonstration of skill in treating patients and having good access to western pharmaceuticals.

Although the government ultimately lacks the capacity to regulate drug sellers, drug shops are increasingly judged (for the most part negatively) against the standards of the formal health sector (MHSDMU 2011). The results of occasional regulatory visits are not restricted to the confines of the reports by regulatory authorities and the civil service. Instead, they become sensational news reports about sales of stolen and expired medicine and dangerous medical practice by untrained drug shop vendors driven by a desire for profit rather than care. These reports generate a powerful narrative at national level of the ongoing work of the state in regulating and monitoring health care providers. The reader is encouraged to evaluate the work of the drug shop vendor as a professional medical practitioner within the formal system and the reports encourage clients to choose drug shops registered with the government who have medical training or to seek care in the public sector.

Among members of the community in our study, however, the ability to choose between registered and unregistered drug shops in Mukono prior to the project was limited. As in other unregulated, unorganized health care markets, the issue of trust, assessment of skills, and maintenance of confidence and legitimacy dominated much of the debate about drug shops (Bloom, Standing, & Lloyd, 2008). Members of the community complained about the difficulty of evaluating health care providers and differentiating between those who were both educated and skilled; and those who had gained sufficient knowledge (working as an auxiliary member of staff in a hospital or as a member of domestic staff in a health workers household) to be able to deceive vulnerable clients looking for care.

## Results

### Joining the project

Within this context the project began its contact with drug shop vendors in Mukono, going to visit those registered with the local district health office. The project had sought to minimize issues surrounding informality by choosing to work with registered drug shop vendors only. With this in mind, on arrival at the shops, project staff anticipated a relatively simple procedure of going through the details, offering the drug shop vendors the chance to join. Although the subjects of the project represented some of the most formalized Ugandan drug sellers, during the focus groups some reported that on being warned (by observant neighbours or those in nearby shops) of the arrival of a project vehicle with recognizable government number plates, their initial response was simply to shut up shop and hurry away.

Those who were recruited into the project were formed into clusters and randomly assigned to the intervention group (where they would receive training, equipment to take a blood sample RDTs, ACTs and rectal artesunate) or the non-intervention group (where they would receive training, equipment to sample blood, ACTs and rectal artesunate). Returning from the training to their drug shops to begin taking blood and providing ACTs, the drug shop vendors reported a significant shift in their perception of their relationship with the state. Membership of the project was described by one as helping her, ‘not to be on risk, because (before) every time whenever you would see a vehicle, Government vehicle passing, you would say maybe they are going to arrest me … I used to fear them very much’. Training certificates from the project were also considered to confer confidence, providing official protection from authorities who would confiscate illegally stocked drugs.

P5: Another thing, this certificate that they gave us, for me on my side I see that it is helping us a lot because even if someone came, you tell him/her that this is the health worker of here. Even if he has never seen you, even if it is someone from the sub county or from the district, the moment you give him this thing (the certificate) like this, he just looks at it and he sees that what this one (the drug shop vendor) does is important and that saves you from a person who would have taken away your drugs.

Focus group with DSVs in the intervention arm who are the second most frequent referrers

### Project training and supervision

In addition to the early visits of the project team to recruit drug shop vendors, visits were made to the drug shops to provide supervision after the initial training (up to three visits over the first two months of the project for shops in both arms) and towards the end of the trial for follow-up of clients. Despite the fact that the project supervisors did not provide comprehensive supervision for the entire duration of the project, members of the community interpreted the ongoing involvement of the project to have provided considerable protection for them from the activities of unskilled drug sellers. For the drug shop vendors, these visits by project staff provided a public demonstration of a new relationship with the state authorities, signalling to potential clients that the provider was competent and offering clients a basis upon which they could trust their claims to expertise.

P7: The other time, some of the people would say, “this health provider didn’t at all go to school or get trained but he simply started (a drug shop)”. He for sometime stayed with someone who was a health provider and this (person) who taught him how to sell panadol and he also did what, started (operating a drug shop)”. But now when they (supervisors and follow up staff) came, they (the clients) got to know that this one didn’t pass through the window (i.e. is qualified). He has some skills and the government knows them.

Focus group with DSVs in the control arm who referred the least often

For members of the community, the involvement of the project in training and supervising the drug shops under the project in the area spilled over into a shift in the overall identity of the drug shop vendor and their position on the margins of the formal health service. Under the project, the drug shop vendors changed from being untrustworthy characters (see also Chandler et al., 2011) who might fool sick and vulnerable clients into thinking that they are experienced health workers and misuse powerful forms of bio-medicine (injections) into legitimate and skilled health workers.

P2: … in the past when you went to a clinic (drug shop), you would tell him (the DSV) ‘I feel I am a bit weak, I feel as if my whole body is broken,’ and he would tell you, ‘ I am going to give you an injection.’ He has not yet known what you are suffering from and has decided to give you an injection. You have not known how I am, okay how I feel. I have just told you that I am weak, but you don’t know what type of weakness, you have not known which side of mine is weak or what but you have right away told me about an injection. But now what has mostly made me happy, that follow up that the Ministry of Health has been doing has so much helped to remove the other thing of ‘I have the experience on panadol (analgesics) , what, let me go and start up a drug shop’. Whereby you don’t know what you are doing and the people you have taken to it also need treatment but you don’t do what, you don’t have full experience on it, that one has so much changed in our drug shops these days.

Focus group with the mothers who had visited the drug shops in the control arm in the past eight months

### Testing blood for malaria in the drug shop

In both arms of the project, involvement in it brought immediate economic benefits: drug shop vendors reported an increase in consultations (which may have arisen due to availability of ACTs at affordable prices and/or increased customer confidence in DSVs), as well as other benefits from improved cash flow: selling RDTs and/or Coartem that had been given free of charge by the project (‘You have helped me to have my income increase because now I can get money that I get from that coartem we get at no cost and then be able to buy other drugs’). Yet surrounding this rather straightforward economic transaction, were the socially and symbolically important aspects of the intervention, the drawing of blood for blood slides and, in the intervention arm also for RDTs. Testing blood was considered to mark a boundary between drug seller and health worker, shifting drug shop vendors into the category of endorsed professional.

P4: … it’s not that we studied much but the patients themselves know that we are real health workers, they no longer know us as people only selling drugs (CN: mmh!) now they know that we also test what … even blood.

Focus group with DSVs in the intervention arm who are the second most frequent referrers

P3: For this thing (RDT) to test this little blood. Before, you know National Drug Authority doesn’t allow a person like us, to say that you get a person and draw blood from him, like you aren’t allowed to inject a person. Still when it comes to this blood, I don’t know how [the project] convinced them to allow this thing but it has helped us a lot and that’s why you see that we are strong now.

Focus group with DSVs in the intervention arm who referred the most

Yet, the testing of blood did not simply signal a symbolic division between drug seller and health-worker, standing in relation to a fixed cultural interpretation of the meaning of blood and the meaning of drawing blood in these spaces. The interaction of the research subjects with the test and objects surrounding the test, the performance of drawing and testing blood in front of the client were critical to the production of the status of the drug shop vendor.

P9: Now we are new, our brains were awakened and are now active. Even when you are working, you are as if you are still in the training, you are preparing this and that, and you go to the patient knowing what you are going to do for him. Because the way you put on gloves, when you have arranged various things and whatever you open is clear. Ehhe (yes) and they also see us, that though we are in drug shops, we are as if we are in health facilities. Only that we lack a uniform (laughter from the other focus group participants).

Facilitator: What do you have to say about that issue of uniforms?

P9: We would all want them. But there is a way he/she looks at you when you bring a tray with all that is there. You open the swabs and they are clear. He may think you are going to pierce using an injection but you use what? (a lancet). And this thing is clear and clean and does not have to spread HIV because the gloves are there and are new. You simply pick them (up). And so, they (the clients) see that we are health workers. This has a way it puts life in us and we feel big, ehhe (yes), you see yourself becoming big. If they give us uniforms, we can be happy, and even the mosquito nets … (laughter from the other FGD participants)

Focus group with DSVs in the intervention arm who refer the most

It is in the form of the RDT that conducting it effectively (according to the training) necessitates the collection of blood, placing it in the test, adding the buffer and waiting for the results. Under the conditions of the project with adherence to good practice taught during training and reinforced during supervision, it also means that gloves must be worn, and a sterile lancet used to pierce the skin. For the drug shop vendors, concerned with their need to demonstrate skill in an on-going way to ensure their authority and legitimacy in the eyes of clients, the benefits of the rapid diagnostic test lies in its ability to enable drug shop vendors to use a variety of visibly medicalized objects, associated primarily with trained, formal sector and skilled health workers, openly at the front of the shop and in front of the client.

In the extract above, much is made of the fact that part of the way in which the RDTs (and the medical products used in conjunction with it) are defined is in relation to injections. Injections have been defined as the object par excellence that allows for institutional belonging to be appropriated by informal bio-medical suppliers around the world (Birungi, 1998; Mogensen, 2005; Pinto, 2004; Reeler, 2000). Our research in Mukono echoes findings in India and elsewhere in Uganda that injections are perceived of by clients and providers as both a potent form of bio-medicine that can raise the status of these marginal drug sellers, and as also risky, both socially and biomedically (Birungi, 1998; Pinto, 2004). In Mukono, among members of the community, the unskilled, overuse of injections resulting in infection (mostly identified as abscesses) was particularly associated with drug shop vendors who made false claims about their skill and knowledge of bio-medical practice. Here the RDT references notions of power and potency through its association with injections and possibly the need to pierce the skin but at the same time, the use of gloves and packaged lancets negate the negative connections between injections and the spread of disease.

### Referring patients from drug shop to health facilities

In contrast to the profound effect of the project in shifting the perception of the drug shop vendors’ position in the formal health service at community level and in the drug shops, this transformative effect of the project at government health facilities and government and mission hospitals in Mukono was much more limited. FGDs with health workers revealed that although some were aware that drug shops were conducting malaria tests and had noticed a decline in the numbers of patients attending their facility for a malaria test, most knew little of these changes.

In many ways this lack of knowledge is unsurprising, as the project’s main activities were outside the formal health service. Yet, the project had aimed to create a system that included the introduction of new documentation through which critically ill clients (including those who had been given pre-referral rectal artesunate) and other non-emergency cases could be referred into the formal health service. The project provided referral forms written in the local vernacular (Luganda) to be completed in triplicate by the drug shop vendors: a copy was to be kept at the drug shop, a second taken by the client to the health-centre and the third copy to be retained by the project as data for the assessment of the referral system. There were two different types of referral form, one for emergency and the other for non-emergency referrals and the drug shop vendors were trained to assess clients for referral and fill in the forms. During the focus groups, and echoing the interpretation of the meaning of the performance of the RDT test in the drug shop, the drug shop vendors recognized the potentially socially transformative nature of the referral form both in the health facility and in the community.

P4: Before we started making these referrals, the health facilities weren’t recognising us. But now, when the health facilities learned that we are also there, and we test blood and we write to them, they get to know that we are also a bit educated (laughter).

Focus group with drug shop vendors in the intervention arm who refer the most

P8: And when you give these patients the forms, they take them to be important and you tell him that when you come back, you bring it to me and I see. Then you can be in position to keep it. He should bring it for you to see, because you, you are his health worker in the village, for him to find value in you.

Focus group with drug shop vendors in the intervention arm who are the second most frequent referrers

These were, however, isolated reports of the apparently positive impact of the completed referral form on the drug shop vendor’s status. Although the majority of health workers agreed that referral from drug shops occurred rarely and should be encouraged to make clinical decision-making easier and more effective at the health facility, the project retrieved only four completed referral forms at the health facilities. The majority of health workers participating in the focus groups had never seen the project referral form and of those that had, many reported that they had discarded it.

P6: I might have seen it [the referral form] but I don’t pay attention. Is this herbalist or others, say?

P5: I used to see it but I thought it was from herbalist so I never paid attention … (all laugh) ...

Facilitator: Why did you think it was from herbalist?

P5: It was written in Luganda so in fact I never expected something from a health worker to come in Luganda.

P3: To add on his, what he has said. I saw like three, but the first one I saw, the handwriting wasn’t all that good and the form was in Luganda. Me, I never bothered to read because I thought the patient was from a traditional personnel and the person has come to the hospital so I started afresh … (all laugh) … because the handwriting wasn’t like for a trained person.

Focus group with health workers in the control arm from a private not for profit hospital

For these health workers, the project referral form was a document out of place at the health facilities. It was not recognizable as the official government referral form, had no Ministry of Health logo on it, was longer than the standard form and moreover was in a language that was associated with traditional practitioners, not bio-medical health workers. As discussions unfolded and the health workers recognized that this form conveyed bio-medical information in Luganda from drug shops to health-centres, the disruptive potential of the project’s referral form was revealed.

P7: I think this form, we are encouraging these unqualified people …

P1: Exactly! It was what I was going to say (laughs)

P9: There is no ‘Gray’s Anatomy’ written in Luganda!

P7: I still stick on my point that this thing being in Luganda. It will encourage my sister down there who stopped in primary school grade 3. (Quoting the form) ‘Write the age, temperature, strength …’

P6: Temperature (correcting P7)

P7: Body temperature (laughter). So that means bring a digital thermometer, put, when you see CC write. So, it’s very easy to educate someone who is not trained in this thing and they refer. I think we will have encouraged more …

P6: … dropouts

P7: (School) dropouts to encroach.

Facilitator: So what change do you think the English one (form) would bring?

P7: The English one? Say for instance, doctor (referring to P9), can help me? You had a view (laughter).

P9: Many of us are trained in English, it is quick for us to read, two, the wordings are very short. And the drugs also (laughs).

P1: Drugs are not written in Luganda; aspirin.

P7: They are not in Luganda.

P8: I think that point is very important. Already we are trying to train, you know this information is enough to train a sweeper in the hospital to start a drug shop. Because, you are telling them what to do. This is information enough to educate them. This thing has data few of us can understand it. It is so bulky. If you get a very sick patient by the time you go through this, the patient may have progressed to worse.

Focus group with health workers from sites with inpatient facilities, serving both arms of the project

Like the drug shop vendors, the health workers recognized that writing on and reading bio-medical forms in the context of a health-centre is a powerful social act (Brown, 2012; Pinto, 2004). Demonstrating skill, authority and belonging to senior members of the bio-medical community, these forms appear to appropriate elements of the identity of the writer and mediate social relationships. Imbuing the form with significant power, the health workers considered that the form acts not only to enable but also encourage uneducated and untrained people to mimic health workers and practice bio-medicine.

The project referral form, materially out of place in the health-centre, facilitates a strong association with the drug shop vendor, carrying with it an imprint of the drug shop vendors’ liminality in relation to the formal system. In the context of the health centre where hierarchies are fixed, authority and power claimed upon the basis of education and language, claims to expertise are closely guarded and regulated. If the expertise of the school ‘drop out’ or ‘hospital sweeper’ are accepted in the guise of a referral form then this would offer a direct challenge to the claims of authority that can be made by formally trained health workers. Situated within the health-centre, the referral form was seen as a threat to the boundaries of the formal health system and the claims to authority of these health workers.

The discussion that unfolded in the focus group, and during patient consultations in which the referral form was produced, worked to deny this challenge to bio-medical authority. The form became a means of reinforcing the marginality of the drug shop vendor, situating them beyond the boundaries of the formal system, an identity that the supervisory visits and use of RDTs in drug shops at community level had challenged. It also offered these health workers an opportunity to demonstrate the legitimacy of their education, knowledge and ability. The hierarchies of the medical system came into full view during the focus group as the clinical officer (P7) gave way to the doctor (P9) to form the narrative of the importance of medical training, use of language and knowledge about drugs. As a direct threat to the identity of the health workers and the basis upon which they make their claims to authority and power, the form had to be recast as a sign of the lack of education, training and ultimately lack of belonging to the formal bio-medical community. The drug shop vendors themselves in both arms of the project were highly aware of this interaction between their referred clients and the health workers in the local health facilities.

## Conclusions

In this paper, we have sought to examine the interface between the project, its constituents and the health care system by providing an analysis of the moment at which the local residents and health workers came into contact with the project’s material products and the subjects of its training and supervisory programme. While the project aimed to improve malaria treatment and reduce risks associated with presumptive treatment by introducing diagnostics into the private sector, the local constituents of the project were also concerned by the way in which it reflected the relationship between the drug shops and the formal health service. Seen from below, the project was not only about the introduction of a new diagnostic technology but also about its capacity to increase the legitimacy of these private sector providers by drawing them closer to the formal sector in a health care system characterized by high levels of unorganized markets, porous boundaries between sectors and a lack of state regulation (Bloom, Standing, & Lloyd, 2008).

Pinto (2004) argues that two sets of questions must be asked of marginal bio-medical providers, the first about how they achieve legitimacy and the second about the global and national conditions that make such work necessary, inevitable but ultimately exclusionary. Drug shops provide the majority of pharmaceuticals used to treat febrile illness in Uganda, a common complaint and sign of dangerous illness. Yet, they do more than this. Well-stocked drug shops and drug shop vendors skilled in the performance of bio-medicine work to keep local desires for pharmaceuticals and access to trained, regulated bio-medical health workers alive in the context of a fractured public health care system in which health workers are often missing and medicine unavailable (van der Geest & Whyte, 1989). Yet in working as mimics, drug shop vendors provide a form of bio-medicine that, at this local level, can only be accepted within the liminal spaces of the drug shop. As soon as the project moved out of the shop and attempted to institute a system of referral that transferred the drug shop vendors’ bio-medical knowledge into the health-centre, the drug shop vendors’ claims to expertise and authority could no longer be tolerated by health workers.

Recent technological developments and policy shifts towards drug sellers in the global health arena are opening new spaces in which drug shop vendors may be able to achieve a much-desired legitimacy on a more consistent basis. Diagnostic tests for malaria, once the domain of the health-centres with access to microscopes and trained personnel have evolved technologically to enable them to spread into more informal spaces such as these Ugandan drug shops. When their use is sanctioned by those in authority these tests take with them much more than the ability to test blood for parasites: they carry and stabilize meaning around the skill, ability and trustworthiness of the person conducting them.

For those wishing to introduce these medically and socially powerful diagnostics into these spaces at the margins of the health system, the regulation of these shops will become imperative and they will need to find a position for drug shop vendors within the hierarchies of the formal system. Without these pieces of the policy puzzle in place, the introduction of RDTs into drug shops in countries like Uganda risks making drug shops much more attractive places to seek care without safeguards in place to protect clientele against other potentially hazardous services sometimes on offer, like injections; nor the ability to take clients with more complex care needs out of the shop and into the health facility.
